# Optimising the emergency-readiness of public access defibrillators across Wales using quality improvement methodology

**DOI:** 10.1136/bmjoq-2025-003585

**Published:** 2026-04-10

**Authors:** Susan Goodfellow, Wendy Hardyman, Jamie Sullivan, Julie Starling

**Affiliations:** 1 The Health Foundation, London, UK; 2 NHS Wales Health Education and Improvement Wales, Cardiff, UK; 3 Cardiff Metropolitan University, Cardiff, UK; 4 Networks & Planning Health Intelligence Service, NHS Wales Executive, Cardiff, UK; 5 NHS Wales Executive, Cardiff, UK

**Keywords:** Quality improvement methodologies, Cardiopulmonary Resuscitation, Prehospital care

## Abstract

Early bystander cardiopulmonary resuscitation and use of automated external defibrillators (AEDs) have been shown to significantly improve survival from out-of-hospital cardiac arrest (OHCA). Public access to AEDs, also known as public access defibrillators (PADs), is hence a critical component for successful emergency bystander intervention.

Wales currently has over 8000 PADs registered on a UK-wide defibrillator network—‘The Circuit’ supported by the British Heart Foundation. This enables emergency services to direct people to the nearest emergency-ready PAD when an OHCA occurs. However, not all PADs are on The Circuit as registration is not mandated, and maintaining PAD fleets in emergency-ready status represents a substantial challenge. Limited research exists regarding the veracity of operational status of PADs in real-world settings, or initiatives which increase numbers of available emergency-ready PADs.

This national quality improvement (QI) project assessed the introduction, spread and scale across Wales of an innovative role, Community Coordinators, funded by Welsh Government through the Save a Life Cymru programme. The project aimed to increase the emergency-ready status of the Welsh PAD fleet by 5% from a baseline of 89% by June 2024. Data from The Circuit supported establishment of baseline measures and ongoing data analytics during the study period (August 2022–July 2024).

The introduction of Community Coordinators increased the proportion of Wales’ emergency-ready PADs from 0.89 to 0.94. Process measures indicated firstly, an increase in registered PADs in Wales from 6415 to 8638. Secondly, the proportion of PADs registered with a PAD Guardian on The Circuit increased from 0.73 to 0.89.

This QI project demonstrated that the problem of PAD non-readiness is complex, multifactorial and dynamic. Networked Community Coordinators to support volunteer PAD Guardians increased the emergency-ready status of the all-Wales defibrillator fleet. This innovative Welsh model could benefit wider UK and international communities.

**WHAT IS ALREADY KNOWN ON THIS TOPIC**

- Limited research exists on the operational status of public access defibrillators (PADs) in real-world settings or of strategic quality improvement initiatives to maintain emergency-readiness of a national PAD fleet.

**WHAT THIS STUDY ADDS**

- The spread and scale of a unique role, regionally networked Community Coordinator, improved the emergency-readiness of a national PAD fleet and enhanced knowledge of the complex system this aimed to improve.

**HOW THIS STUDY MIGHT AFFECT RESEARCH, PRACTICE OR POLICY**

- The findings emphasise the value of employing networked Community Coordinators to support volunteer community guardians of PADs, and the potential for replication, wider spread and scale of this role to other healthcare contexts. Policymakers could consider mandating the registration of new PADs on a single national defibrillator network to ensure that emergency services are aware of their existence, location and emergency-ready availability.

## Problem

Wales, UK has a lower estimated 30-day survival rate from out-of-hospital cardiac arrest (OHCA) (approximately 5%) than other UK nations^1^ and many European countries, where reported survival rates range from 3.5% to 35%.^2^ The impact of this problem is avoidable harm in the Welsh community.

Although the National Health Service in Wales is publicly funded and free at the point of delivery (for those ordinarily resident in Wales), cardiovascular disease (CVD) places huge financial demands on this service (£770 million a year).^3^ Indeed, CVD accounts for 27% of all deaths in Wales, with deprived areas exhibiting higher rates of CVD mortality and morbidity.^3^

Making improvements to OHCA outcomes is complex and requires working at scale, as any person in Wales (population circa 3.19 million) could potentially suffer an OHCA. This unpredictable medical emergency can occur anywhere, anytime, often far from trained medical support. 80% of OHCA events occur within the home.^1^

No UK legislation exists regarding mandatory provision of public access defibrillators (PADs) in public places; mandatory registration of PADs on a single, national database; or restrictions regarding who can use PADs in the event of an emergency such as OHCA.^4 5^ There are no reported cases in the UK of lay-rescuers being successfully sued for helping in an emergency situation.^4^

Survival chances and outcomes from OHCA increase if the first three links in the chain of survival occur in an efficient, effective and timely manner: early recognition and call for help, early cardiopulmonary resuscitation (CPR), early defibrillation.^1^ Attitudinal data from sequential population surveys in Wales show the general public lack confidence to intervene as bystanders (ie, provide early CPR and early defibrillation) in the event of an OHCA.

We had access to data from ‘The Circuit’^6^ (a live database), regarding the total number of registered PADs in Wales, and their availability for use. We know from this data that many existing PADs are unavailable for use during OHCA, which is likely to reduce both the effectiveness of bystander intervention and OHCA survival rate. We understood from work undertaken on improving OHCA outcomes in North Wales that significant numbers of PADs were not registered on The Circuit, so were not included in The Circuit data.

At the start of this project, Wales had 6415 PADs registered on The Circuit. Of these, a proportion of 0.89 was available. We had an idea of ‘what good looked like’, based on a pioneering project to improve OHCA in North Wales, which achieved a proportion of 0.94 PADs emergency-readiness locally.

The project aimed to increase the emergency-ready status of the Welsh PAD fleet by 5% from a baseline of 89% by June 2024.

## Background

Promoting early bystander intervention (CPR and defibrillation) forms part of a multi-layered strategy to increase OHCA survival, which includes optimising availability, reliability and usability of PADs to communities.^6 7^ However, maintaining PAD fleets in emergency-ready status represents a substantial challenge.^7^,^13^ In Wales and the wider UK, PADs have been funded through multiple sources such as government initiatives,^5^ charitable organisations, community fundraising and corporate sponsorship. These devices are sold by multiple sources, which raises challenges regarding mapping of all PADs placed in communities.^6^ Although PADs may get placed in areas of high footfall (eg, shopping centres, train stations, sports clubs) international evidence suggests PAD placement in communities has traditionally been haphazard, not centrally co-ordinated, with placement decisions left to PAD owners.^7^

Responsibility for maintenance mainly falls on owners of PADs, with widespread variation internationally regarding the existence of ‘enabling legislation’^12^ for mandating both provision and regular maintenance of these potentially lifesaving devices. Functionality of PADs in real-world settings is currently an under-researched area.^9^ Although studies and campaigns have raised awareness regarding how to use and where to locate PADs, these have not focused on both ‘how’ and ‘if’ these devices are functional over more extended timeframes.^13^ It has been argued that use of technology such as PADs places enormous demands on public engagement for both use and maintenance, with such activities requiring both social support and technical infrastructure support.^13^

Lack of coordinated nationwide efforts, national guidance or strategy to install, maintain and communicate the locations of accessible and emergency-ready PADs has potential to lead to harm from OHCA. This concern is echoed in a recent International Liaison Committee on Resuscitation Scientific Statement^7^ regarding optimisation of OHCA outcomes. This statement outlines: poorly maintained PADs pose a potential threat to life in an OHCA event; variation exists in the quality of maintenance of PADs in real-world settings (ie, lack of associated individuals responsible for maintenance, no formal plans for either maintenance or replacement) and recommends regular checking of PADs in line with manufacturer guidelines to ensure emergency-readiness at all times.

In Wales, PAD owners are encouraged to register their devices on British Heart Foundation (BHF) The Circuit.^6^ This single, national defibrillator network allows individuals or organisations to record information regarding PAD location, accessibility and availability, synchronised with ambulance dispatch systems to enable real-time information on the nearest available PAD. The Circuit issues time and event triggered email alerts to community volunteers, acting as PAD Guardians, to regularly check PAD functionality/availability and update records.^6^ The Circuit was rolled out from 2019 to Welsh Ambulance Services University NHS Trust (WAST).

Adopting strategies to support community resilience to maintain the emergency-readiness of PADs, as well as capacity to intervene, may improve OHCA survival rates. Save a Life Cymru (SaLC) is a programme aiming to improve and strengthen links in the chain of survival from OHCA in Wales. SaLC defined three primary drivers in the community to improve OHCA survival: increased capability and capacity to deliver CPR; population behavioural change to improve confidence to intervene at OHCA, and increased capability and capacity for defibrillation. Work progressed for each driver during the course of this project, but for this report, we concentrate only on how we improved the emergency-readiness of the all-Wales PAD fleet.

Previous work in North Wales, The North Wales Community Out-of-Hospital Cardiac Arrest Project (NWP), involving SaLC programme team members (prior to commencing SaLC posts), revealed substantial problems with defibrillator fleets in Betsi Cadwaladr University Health Board (BCUHB), the largest Health Board in Wales. Assessment of BCUHB PAD sites in 2018 revealed three fundamental issues: cabinets which were waterlogged, rusted or broken; defibrillators which were damp, in need of replacement/out-of-date, or included used pads and batteries; and some PAD sites were not registered with the WAST, so they were not deployed by emergency call-takers.

Responding to these challenges, an innovative role—Public Access Defibrillator Support Officer (PADSO)—was established. The PADSO was tasked with engaging with communities and managing PAD sites in BCUHB. Learning from NWP informed the development of the project reported in this paper, the testing, spread and scale of the PADSO role by employing six SaLC Community Coordinators across Wales.

The introduction of Community Coordinators (referred to hereon as Coordinators) is a unique national initiative, which can enhance emergency-ready status of PADs and warrants exploration of impact through quality improvement (QI) methodology.^14^ We are not aware of similar initiatives adopting this approach reported in extant literature.

## Measurement

### Outcome measure

The proportion of PADs registered on The Circuit as emergency-ready.

Operational definition: the proportion of Welsh PADs registered on The Circuit as emergency-ready, divided by total PADs in Wales, calculated at 09:00 every Tuesday.

Rationale: Optimum measure of preparedness of Welsh PADs for emergency deployment.

NWP represented ‘what good looked like’ with a proportion of 0.94. As the fleet of Welsh PADs increased during the project, we used p-charts for accuracy with different sized cohorts.

Three process measures linked our observed outcomes to SaLC interventions:

1. The number of PADs registered on The Circuit.

Definition: Total number of PADs registered in Wales on The Circuit every Tuesday at 09:00.

Rationale: PADs not registered on The Circuit would typically be unknown to 999 call handlers, so effectively unavailable when call handlers signposted bystanders to their nearest PAD.

1. The proportion of PADs registered with a Guardian.

Operational Definition: Proportion of Wales PADs registered on The Circuit with a Guardian, calculated from total with a registered Guardian divided by total number of PADs and, every Tuesday at 09:00.

Rationale: Learning from our ‘Understanding the Problem Pareto analysis’ highlighted the main causes of ‘offline’ PADs related to Guardians. Assigning Guardians would ensure sustainability of PADs emergency readiness.

Validity and reliability for two measures above:

The Circuit is the most comprehensive database for UK PADs location and status. Despite the dynamic data, with PAD status changing every few seconds, we considered our measures to be valid and reliable, as most were based on data extracted from The Circuit.

1. Number of PAD site visits by Coordinators during routine work.

Operational Definition: From Coordinators’ daily worksheets, the number of site visits made per day per Coordinator between 1 February 2023 and 31 June 2023, presented as aggregate data for this period.

Rationale: A PAD site visit was considered the most efficient method of assessing a PAD for emergency readiness, given the known multiple reasons for PADs being ‘offline’.

Validity and reliability: This is a valid data source but may be prone to human error during both data input and extraction stages.

Balancing measure: We chose to estimate the environmental cost—in metric tonnes of CO2 per annum—of the miles driven by Coordinators while visiting PAD sites.

## Design

Stakeholder analysis showed groups engaging with the SaLC programme included: colleagues from the NHS, key stakeholders, community volunteers and patients/relatives with lived experience of OHCA. Patient and public involvement in this project was limited to engagement as essential stakeholders.

Our project team included: from SaLC Team—Clinical OHCA Programme Manager, six Coordinators, Postdoctoral Research Fellow, Communications Lead; and from Wales Cardiovascular Network—QI adviser, Advanced Health Intelligence Analyst, and a Project Support Officer. BHF The Circuit and WAST colleagues kindly engaged throughout.

This project focused on change ideas to improve PAD emergency readiness, within our third driver of ‘Improving capability and capacity for community defibrillation’.

**Figure 1:**
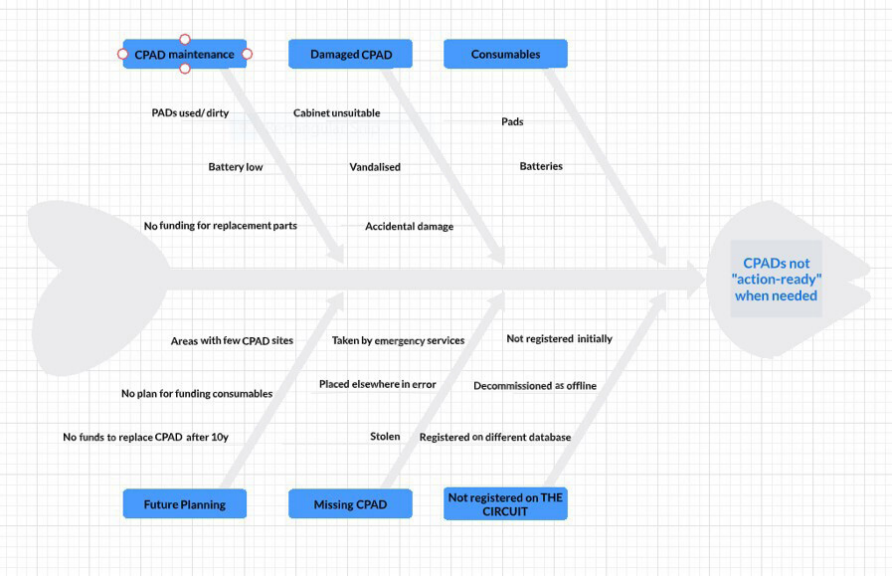
Fishbone diagram of reasons why a PAD may not be emergency-ready. CAPD, community public access defibrillator; PAD, public access defibrillator.

Time spent understanding the problem and building on lessons learnt during the NWP enabled us to define an ‘emergency-ready’ PAD: it must be registered on The Circuit, with working batteries and new defibrillator pads and stored within a heated weatherproof cabinet. Maintenance devolves to a Guardian who replaces consumables and re-instates the PAD promptly after use. The Fishbone diagram (Figure 1) demonstrated 17 reasons why a PAD may not be emergency-ready.

**Figure 2:**
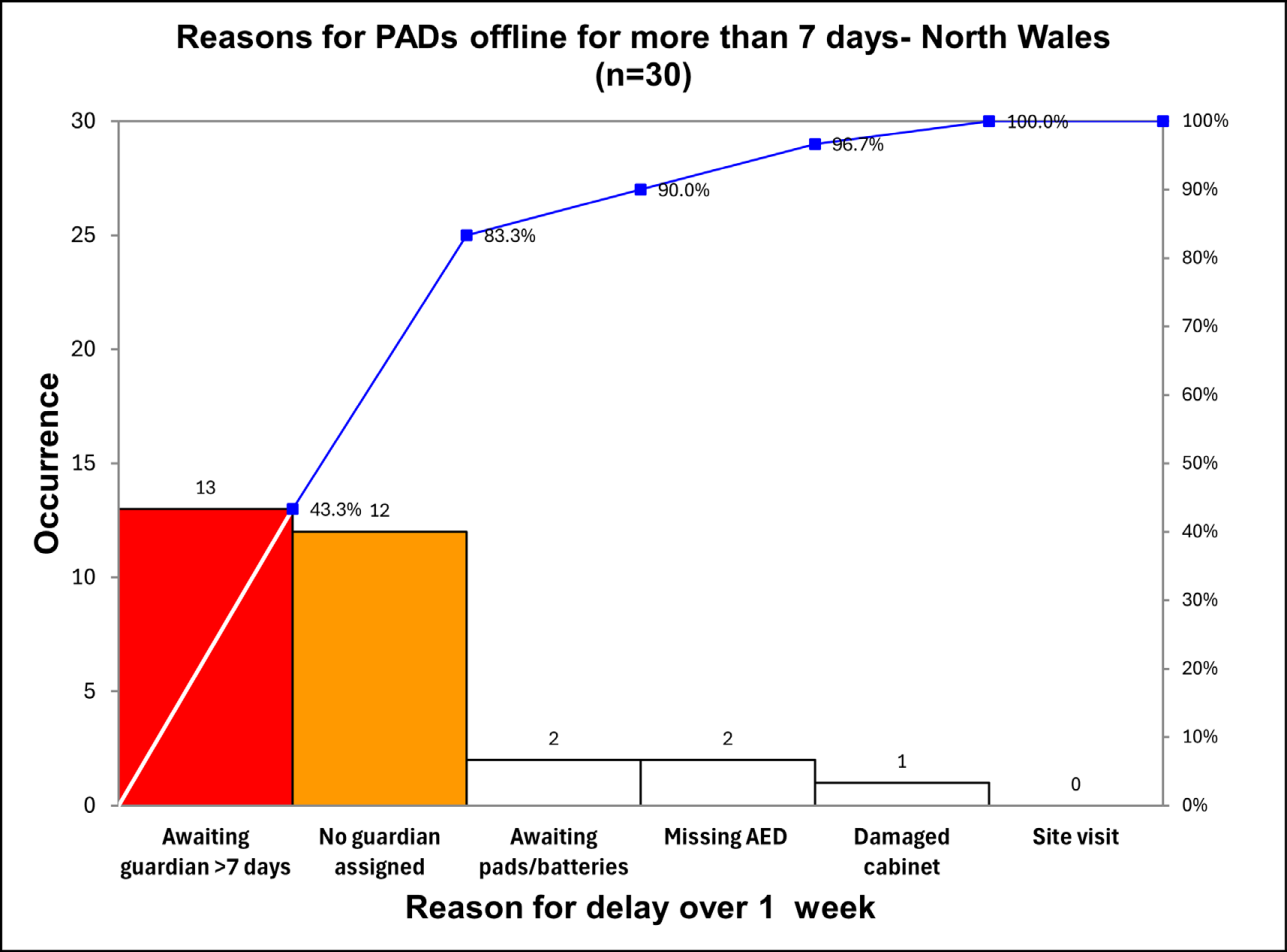
Pareto chart of reasons why a PAD is offline for 7 days or more. AED, automated external defibrillator; PADs, public access defibrillators.

Other QI tools to understand the problem included a Pareto analysis (Figure 2). Pareto showed the dominant causes of PADs non-emergency ready for 7 days or more, related to PAD guardianship—either Guardian not responding to The Circuit alerts, or no assigned Guardian.

To improve emergency readiness of the whole Wales PAD fleet, we trusted the coordinators to act initially on any of the seventeen known causes of non-emergency readiness during PAD site visits.

Next, we reduced waste in the system: those PADs effectively hidden from 999 call handlers, as not registered on The Circuit. Coordinators covering different regions of Wales were uniquely placed for this task, using local knowledge to identify PADs that had not been registered at the time of installation.

Finally, the Coordinators focused on appointing new Guardians and supporting existing Guardians, including the option to extend guardianship to multiple PADs. Support included raising awareness regarding requirements of The Guardian role and signposting to CPR/defibrillation familiarisation or training resources on request.

Our study design developed based on local intelligence provided by the newly-appointed Coordinators. A rigid QI approach based on the results of our Pareto analysis would have first focused the Coordinators on assigning PAD Guardians.

## Strategy

Our strategy was to successfully test, spread and scale the innovative PADSO role in North Wales, using QI methodology and continuous data over time, presented and analysed using statistical process charts to support and inform our next steps.^15^ We were aware of the lessons published in The Spread Challenge, in particular: “Successful implementation may require adaptation of the intervention or a long journey to build new relationships, shift the prevailing team culture or develop new skills.”^14^^(p. 5)^

Several aspects of the work were outside our control: the Welsh Government funded the appointment of six Coordinators across Wales simultaneously, meaning we were unable to spread by scaling up gradually, as we might have chosen to do. These new Coordinators (which included the former NWP PADSO) were appointed nationally but worked regionally (rather than within defined National Health Service (NHS) Health Board boundaries), meaning we could not rely on existing data and knowledge of Health Board patient demographics, culture, staffing etc.

Our change hypothesis: using the audit data outcomes of the NWP as a starting point, switching to dynamic data would better support our learning from Coordinators adopting the North Wales innovation regionally. In turn, this would optimise the gains in PADs emergency readiness across Wales in a sustainable manner. Data from defined outcome and process measures, including The Circuit data, would assist our Plan Do Study Act (PDSA) analysis.

**PDSA 1**: Coordinators conducted site visits to local PADs

Change hypothesis: We believed site visits were the best way to establish and correct the reasons why PADs were not emergency-ready, because the fastest and most sustainable improvements would be achieved by allowing Coordinators to use their subject matter knowledge.

Change strategy: Coordinators were given a clear aim of enabling each PAD in their region to become emergency-ready, and they recorded actions taken to achieve this in each case.

Learning: 17 potential causes were found for PADs being ‘offline’ (Figure 1). One or several applied in each case. This PDSA took months, due to the total number of PADs in the Wales fleet, the geographical challenges of site visits in rural areas, and multiple demands on coordinators’ time within their new roles. We understood the complexity of sustaining an emergency-ready PAD fleet and decided to focus on the ‘quick win’ of registering all PADs on The Circuit during the next PDSA.

**PDSA 2:** Adapted from PDSA 1—Coordinators registered all PADs on The Circuit.

Change hypothesis: We believed that converting existing ‘offline’ PADs to emergency-ready status was the most cost-effective way of increasing PAD availability. Focus on waste in the system—PAD devices are expensive to purchase, not always registered on The Circuit at time of installation, and maintenance costs are not always provided.

Change strategy: Coordinators used Circuit data, cross-referenced with geographical data on PAD sites to identify ‘offline’ PADs that were solely due to not being registered on The Circuit.

Learning: The main reason PADs were not registered on The Circuit was due to omission at the time of PAD installation.

**PDSA 3:** Reduce ‘orphaned’ PADs (those without a Guardian).

Change hypothesis: Pareto analysis (Figure 2) demonstrated the importance of responsive assigned Guardians. We believed increasing the proportion of PADs with Guardians would improve the sustainability of emergency readiness across Wales by reducing reliance on coordinators and sharing the role with motivated local volunteers. We predicted PDSA 3 would be more challenging than 1 and 2, as it involved modifying human behaviour among community volunteers.

Change strategy: Determine Guardian status from Circuit data and assign Guardians where needed.

Learning: Coordinators learnt reasons why Guardians might be unresponsive: communication difficulties between The Circuit and Guardians; emails going to junk mailbox, and Guardian preferences for alternative modes of communication. Some Guardians were unable to continue their role but had not communicated this to The Circuit.

## Results

**Figure 3:**
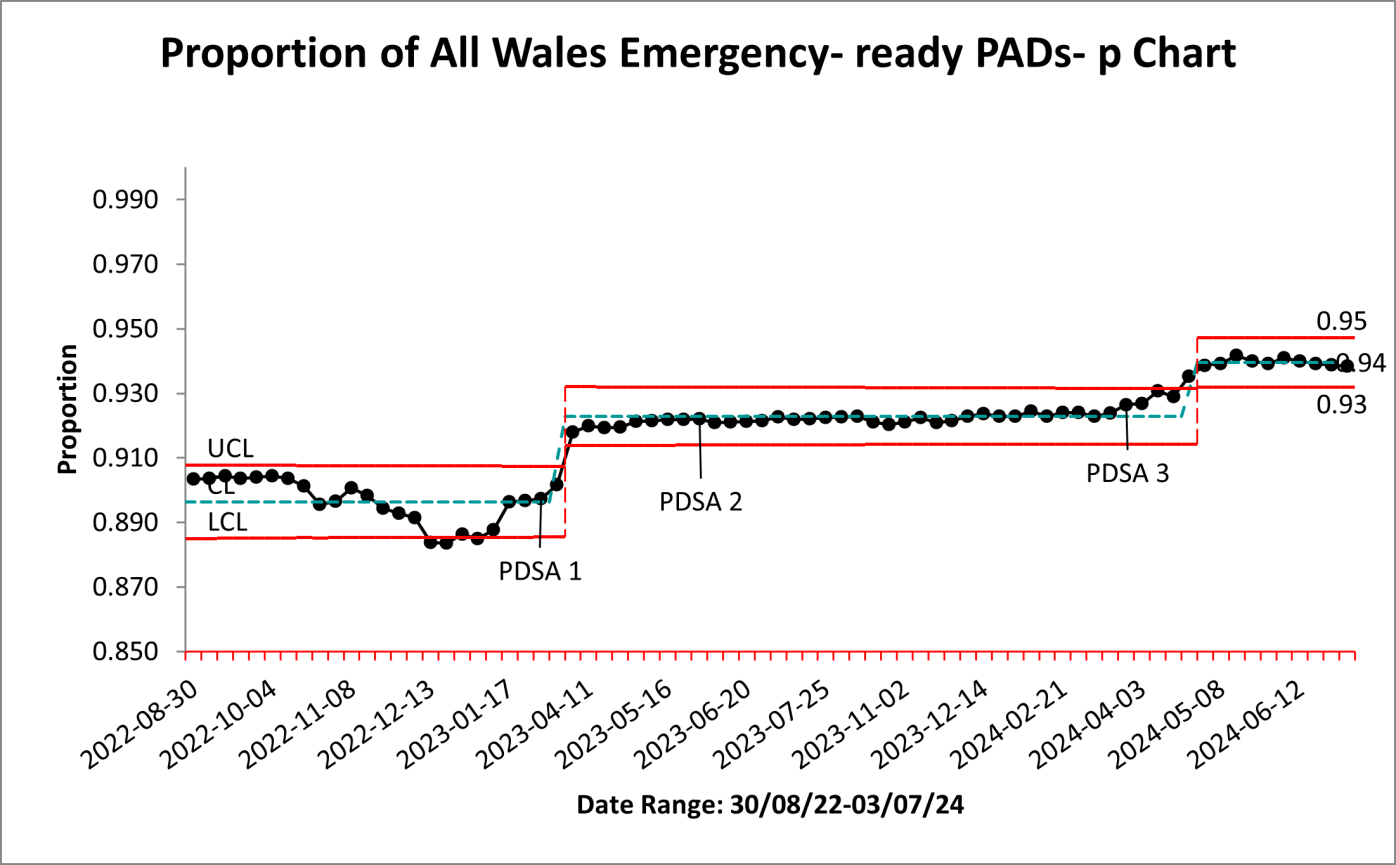
p-chart of proportion of Wales PADs which are emergency-ready. CL, Centre Line; LCL, Lower Control Limit; PDSA, Plan Do Study Act; PADs, public access defibrillators; UCL, Upper Control Limit.

We achieved our aim: the proportion of Wales emergency-ready PADs (Figure 3) increased by 5.6% from 0.89 to 0.94 between August 2022 and July 2024. We chose a p-chart to plot the data, due to variation in subgroup size of total PADs fleet.

Considerable variation in emergency readiness between October 2022 and January 2023 suggested special-cause variation in the system. This was because the experienced North Wales Coordinator was absent for several months shortly before the appointment of additional Coordinators; he supported the induction of those Coordinators, leaving less time to monitor his local PADs.

Once all coordinators became fully operational (February 2023) the emergency-ready PAD proportion shows a shift upwards, coinciding with PDSA 1.

PDSA 2 has a minor impact on the data. Analysis of PDSA 2 with coordinators indicated the action of registering PADs on The Circuit (PDSA 2) was integral to initial site visits (PDSA 1). Consequently, PDSA 2 became a ‘mop-up’ exercise only for new PAD sites—a small proportion of the fleet.

PDSA 3 shows an additional shift of improvement (April 2024) due to assigning Guardians to orphaned PADs. We believe this demonstrated the importance of the Guardian role in sustaining an emergency-ready PADs fleet.

### Process measures

**Figure 4:**
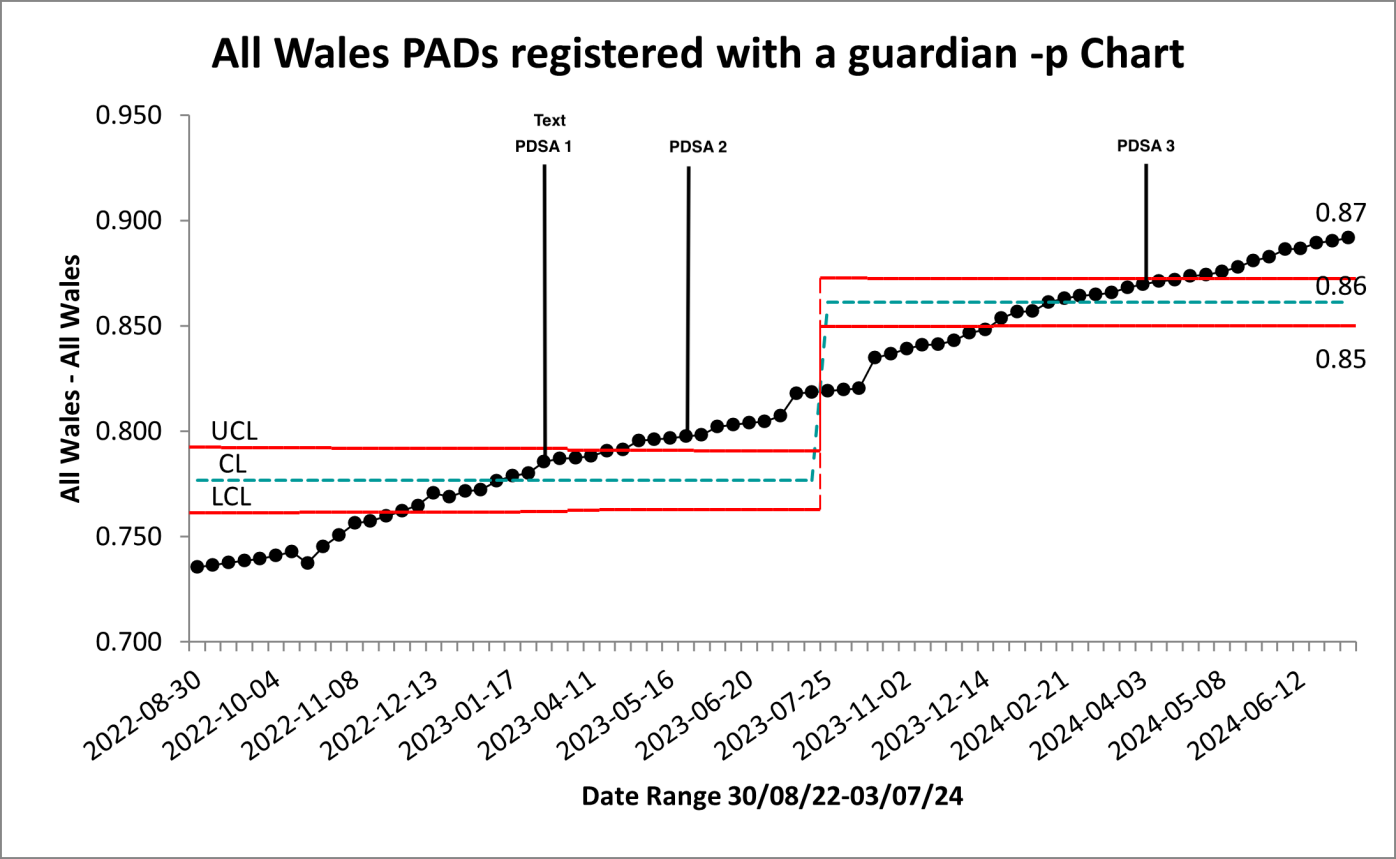
p-chart of proportion of all Wales PADs with an assigned guardian. CL, Centre Line; LCL, Lower Control Limit; PDSA, Plan Do Study Act; PADs, public access defibrillators; UCL, Upper Control Limit.

The number of PADs registered on The Circuit across Wales increased from 6415 to 8638 during the project (Figure 4). The Welsh Government donated 500 new PADs for SaLC to distribute, the remainder of the increase resulted from this project, as numerous existing PADs were registered on The Circuit and lost PADs were found/reinstated. Note the 8-week data gap annotated on this c-chart coincides closely with PDSA 1, giving the appearance of an improvement shift. The data gap requires us to interpret this signal of improvement with caution. However, the increase of 2223 PADs registered throughout the project far exceeds the 500 donated, indicating success by the SaLC team.

**Figure 5:**
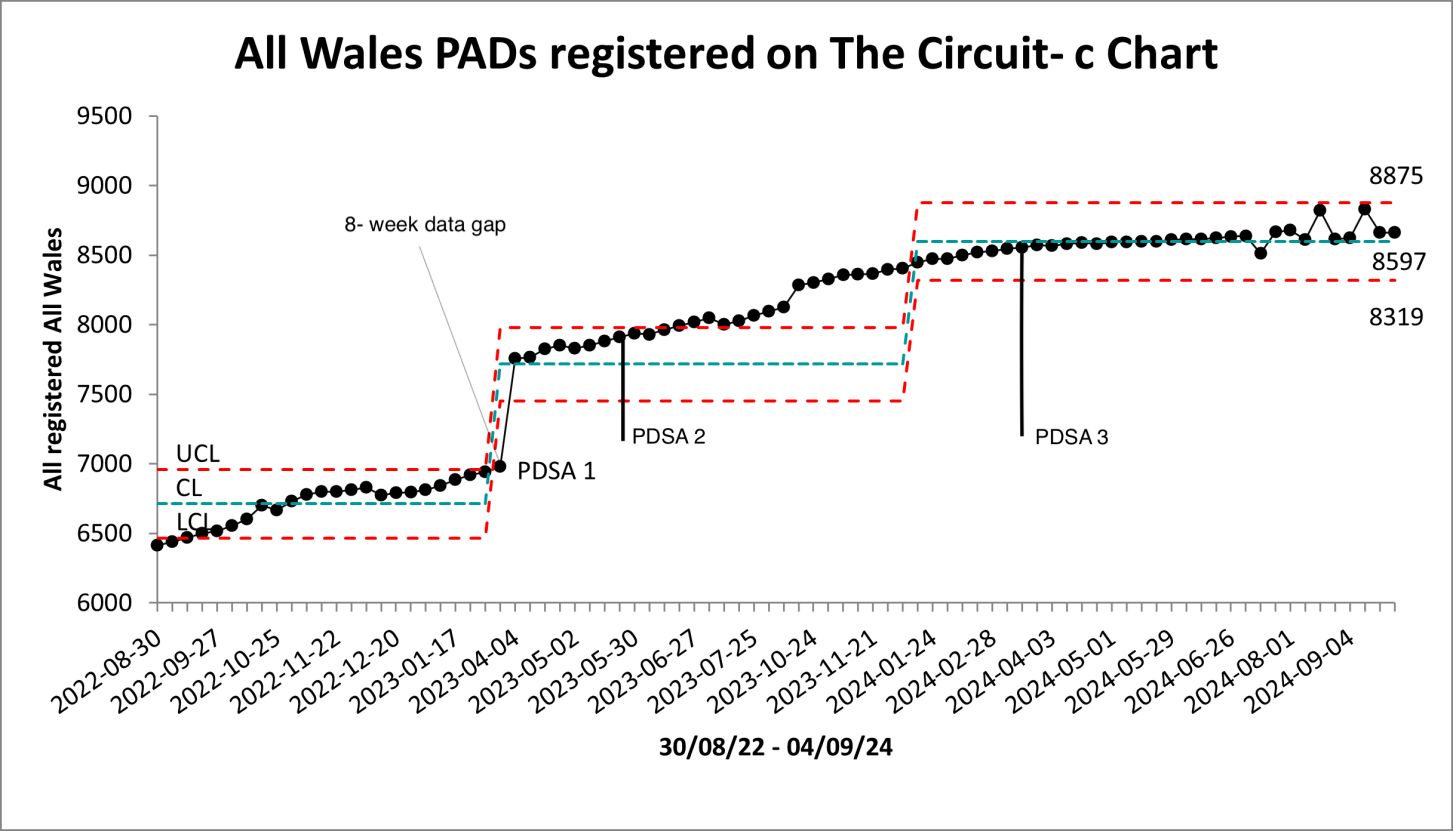
c-chart of count all—Wales PADs. CL, Centre Line; LCL, Lower Control Limit; PDSA, Plan Do Study Act; PADs, public access defibrillators; UCL, Upper Control Limit.

The proportion of PADs registered with a Guardian across Wales (Figure 5) increased from 0.73 to 0.89, showing steady improvement after the appointment of six Coordinators.

275 Coordinator PAD site visits were completed between February and June 2023.

Completed tasks included 107 PADs reinstated after deployment, 16 newly-registered PADs, 43 newly assigned PAD Guardians, 6 missing PADs returned and 103 routine PAD checks.

### Balancing measure

SaLC Scope 1 emissions increased during the project: Coordinators drove 35 866 km per annum work-related kilometres in their own cars, equivalent to approximately 1.4 metric tonnes of CO2 per Coordinator. By comparison, the average UK domestic car use creates 1.35 metric tonnes CO2 per person per annum.

We considered the validity and reliability of our data to be good. Following the data-sharing agreement with The Circuit (August 2022), Wales’ data were extracted directly from The Circuit dashboard at the same time/day each week and copied into SaLC dashboard without manipulation to reduce error. Data signals were then analysed and potential anomalies discussed with the QI team.

There were occasional data gaps outside our control, the longest being 8 weeks from 15 August 2023.

## Lessons and limitations

A highlight of this project was the successful development, spread and scale of the innovative Coordinator role from one Health Board to pan Wales, despite the challenges of different environments, population density and language.

A major enabler was our data-sharing agreement with The Circuit, which granted access to continuous reliable data for our measures. This professional relationship was mutually beneficial and facilitated The Circuit colleagues adopting suggestions to improve The Circuit for end-users.

We learnt about the complexity of the system we were trying to improve and the dynamic nature of PAD emergency-readiness, due to frequent deployment of devices and multiple factors contributing to emergency-readiness of any PAD.

The commitment of our SaLC Coordinators and volunteer PAD Guardians was another highlight and source of learning.

Some assumptions had to be made during data gaps in The Circuit data downloads (which were outside our control).

During data analysis, we detected no significant additional signals of improvement from PDSA 2. In PDSA 1, we empowered the Coordinators to correct every PAD defect during each site visit, including registering an unregistered PAD on The Circuit if indicated. This decision compromised the rigour of separating the PDSA 1 (PAD site visits) from PDSA 2 (registering PAD on The Circuit), but it prioritised patient safety by reinstating PADs as soon as possible—a pragmatic decision made as we learnt about the complexity of maintaining an emergency-ready fleet of PADs.

We had reasons for adopting an alternative, ‘real-world’ approach: Conducting a QI project in a real-world community setting proved challenging, and it was not ideal that each PDSA took months. The complexity of the system and the number of variables outside of our control meant we had to combine testing and implementation methodology in this pan-Wales project.

If we were to undertake the project again, a phased development then ‘Spread and Scale’ strategy would have allowed us to incorporate incremental learning about this complex system for each regional Coordinator appointment.

We understood early on the importance of the Guardian role and the need for others to support and deputise for Guardians on occasions. Guardians have other demands on their time and may be unable to continue the role indefinitely. Different limitations apply to the Coordinator’s role, which only covers core working hours. This combination of factors could lead to the Swiss Cheese model^16^ of multifactorial failure.

In future PDSAs, we aspire to evaluate change ideas to support the Guardian and Coordinator’s roles:

First, improve modes of communication between The Circuit and Guardians, for routine and emergency alerts.

Second, consider recruiting from allied roles to manage those occasions when Guardians and Coordinators cannot respond to The Circuit alerts.

## Conclusions

Maintaining the emergency-readiness of PADs is an ongoing international challenge, with variation reported regarding the existence of ‘enabling legislation’^12^ to mandate both provision and regular maintenance^12^ of these potentially lifesaving devices. In contrast to other research, we iteratively evaluated and demonstrated a strategic pan-Wales initiative to improve PAD fleet emergency readiness. The emergency-ready proportion of PADs across Wales reached a mean of 0.94, achieving our project aim.

We demonstrated that the problem of PAD non-readiness is complex, multifactorial and dynamic; also, the dominant reasons for PAD emergency-readiness in Wales relate to the efficacy of the Guardian model, see Pareto analysis (Figure 2). We showed the importance of appointing Guardians for every PAD, of effective communication with Guardians, and the added value of networked Coordinators to support Guardians in their role. We did not find evidence of similar initiatives being undertaken in the extant literature, or details of similar QI projects conducted in the ‘real-world setting’ in which this improvement project was located. Given this, we propose that this QI project enhances current knowledge in this field of work.

### Sustainability

We have considered sustainability in terms of environmental and social impact, and in terms of a viable service to maintain a national emergency-ready PAD fleet. A detailed economic analysis is beyond the scope of this project.

We believe the environmental impact of 1.4 metric tonnes of CO2 per Coordinator could be offset by reduced Scope 2 and 3 CO2 emissions, created by reduced requirement for newly manufactured PADs as we reinstate unregistered, misplaced and unusable devices, and by reduced turnover of PADs by better maintenance of the existing PAD fleet. As more Guardians are appointed, living close to their PADs, Coordinators may need to do fewer site visits, and their total mileage should be reduced.

We suggest that networked Coordinators provide an additional layer of infrastructure that is, ‘social support’, facilitating the technical aspects of registration, maintenance and emergency-readiness of devices on The Circuit. We believe other ambulance services in the UK have adopted some of the learning regarding the introduction of Coordinators and developed similar roles to the SaLC coordinators, indicating wider spread and scale.

We have improved the PADs fleet operability with a sustainable service in mind; SaLC Coordinators have permanent employment contracts and have developed considerable professional networks regionally to support their work. Our data sharing agreement with The Circuit enables us to continuously monitor our impact.

### Next steps

To improve capability of defibrillation across Wales further, we aim to optimise the locations of PADs by mapping the accurate locations of previous OHCA events with known locations of PADs. This would enable us to advise on placement of new devices and review the locations of 8638 existing PADs.

To explore how to add additional layers of infrastructure (eg, organisation-based support, professional support) to minimise Guardian fatigue and maintain the emergency-readiness of PADs on a sustainable basis.

To explore the possibility of mandating registration of all new defibrillators on a single, national and dynamic defibrillator network such as The Circuit.

## Data Availability

Data are available on reasonable request.
